# Antifungal and Antibacterial Effect of Propolis: A Comparative Hit for Food-Borne *Pseudomonas*, Enterobacteriaceae and Fungi

**DOI:** 10.3390/foods9050559

**Published:** 2020-05-02

**Authors:** Leonardo Petruzzi, Maria Rosaria Corbo, Daniela Campaniello, Barbara Speranza, Milena Sinigaglia, Antonio Bevilacqua

**Affiliations:** Department of the Science of Agriculture, Food and Environment, University of Foggia, 71122 Foggia, Italy; leonardo.petruzzi@unifg.it (L.P.); mariarosaria.corbo@unifg.it (M.R.C.); daniela.campaniello@unifg.it (D.C.); barbara.speranza@unifg.it (B.S.)

**Keywords:** growth index, viable count, growth kinetic, biostatic effect

## Abstract

Propolis is a natural brownish resinous substance collected by honeybees (*Apis mellifera*), with a documented bioactivity against many microorganisms. In this study, the activity of propolis was investigated using some strains of *Pseudomonas* spp., Enterobacteriaceae, *Lactobacillus plantarum*, yeasts (*Saccharomyces cerevisiae* and *Debaryomyces hansenii*) and *Fusarium oxysporum*. Two approaches were used (a modified microdilution protocol and viable count), and the microorganisms were inoculated at two levels (low or high inoculum). The antimicrobial effect of propolis relies upon several factors, like the kind of microorganisms (for example *S. cerevisiae* was more resistant than *D. hansenii*, while *Lactobacillus plantarum* was never affected), the cell concentration (at high inoculum higher amounts of propolis were required for an antimicrobial action), and the mode of action (a delay of growth rather than a complete inhibition). The results of this paper point out, for the first time, the antimicrobial activity of propolis against some spoilers, with a focus on the possible effect; thus, they could be the background to designing an effective tool to prolong the shelf life of foods.

## 1. Introduction

Products of natural origin have been used in traditional medicine for throughout history and represent a potential source of new drugs. Propolis is an example of such a remedy with an interesting antimicrobial activity known since the time of ancient Egyptians and Greeks [1].

The antimicrobial activity of propolis has been extensively reviewed by different authors; it is well known that it is able to inhibit and/or control the growth of a wide range of microorganisms, either Gram positive (*Listeria monocytogenes*, *Staphylococcus* spp., *Streptococcus* spp., *Bacillus* spp.) or Gram negative bacteria (*Escherichia coli*, *Klebsiella pneumoniae*, *Enterobacter cloacae*, *Pseudomonas aeruginosa*), as well as yeasts and molds (*Candida* spp., *Aspergillus* spp., *Penicillium digitatum*, *Saccharomyces cerevisiae*, *Cladosporium* spp., *Trychophyton* spp., *Alternaria alternata* and *Fusarium oxysporum*) [2,3,4,5,6,7,8]. Propolis is composed of more than 300 different components, like polyphenol (flavonoids, phenolic acids and esters), phenolic aldehydes and ketones. The percentage of these substances is as follows: resins and vegetable balsam 50%, Bee wax 30%, pollen 5%, essential and aromatic oils 10%, and some other substances which include organic compounds as well [9]. The composition is affected by the extraction methods; it is generally produced through an ethanol-extraction, although some steps (like maceration) are variable [10].

The bioactivity relies upon different factors, like (a) origin: European propolis possess a different range of bioactivity than Brazilian or Korean ones, due probably to the different qualitative composition in phenols; (b) the strain, as the effect is strongly strain-dependent; moreover, the inhibition of Gram negative bacteria is controversial; (c) the protocol used to assess in vitro bioactivity, due to the low solubility of some extracts; (d) the use in laboratory media or in foods, as it has been reported that the essential oils and plant extracts can interact with food components; (e) the use of propolis as food ingredients or loaded in a coating [11,12,13,14,15,16,17].

Generally, the antimicrobial activity of propolis has been assessed through the viable count and the results reported as the decrease in cell count compared to the control; however, this approach has a drawback, in that is it is not possible to pinpoint an effect different from the biocidal one.

The effects of plant extracts could be reversible and act on the biomass rather than on the viable population; this effect was reported for different extracts and phenol-related compounds at low or sub-inhibitory concentrations [18,19]. Thus, we propose two protocols to study the bioactivity of propolis in lab media: the classical one based on the viable count at high concentrations and the Growth index approach for relatively low amounts (200–1000 ppm) to build a comparative hit on the resistance/susceptibility of some microorganisms to propolis as a prodromal step for application in food processing.

## 2. Materials and Methods

### 2.1. Microorganisms and Media

Strains and their source are listed in Table 1. The following media were used: (i) Yeast Peptone Glucose broth and Agar (YPG) (yeast extract, 10 g/L; bacteriological peptone, 10 g/L; glucose, 20 g/L; agar, 12 g/L) for yeasts; (ii) Potato Dextrose Agar (PDA) for *Fusarium oxysporum*; (iii) MRS broth and Agar (Oxoid) for *Lactobacillus plantarum*; (iv) Nutrient broth and Agar (Oxoid), for *Pseudomonas* spp. and Enterobacteriaceae. PDA, MRS broth and Agar, and Nutrient broth and Agar are commercial products (Oxoid, Basingstoke, UK).

### 2.2. Propolis

Propolis from an Italian pharmaceutical factory was used throughout this study; the concentration of phenolic compounds was about 30%. Stock solutions (20,000–300,000 ppm; these concentrations, as well as those reported in the following sections, were for the amounts of propolis in the solution) were prepared in a hydro-alcoholic solution (1:1); ethanol (96%) was used as alcohol. One-hundred or two-hundred µL of stock solutions were added to the samples in order to achieve the desired concentrations (from 200 to 3000 ppm).

### 2.3. Growth Index of Bacteria and Yeasts

Aliquots of optimal media (20 mL; YPG broth for yeasts, Nutrient broth for *Pseudomonas* spp. and Enterobacteriaceae, MRS broth for lactic acid bacteria) were supplemented with 200 µL of stock solutions to attain a final concentration of the extract ranging from 200 to 1000 ppm and inoculated to 3 log or to 5 log cfu/mL. Two kinds of controls were prepared: (i) media + hydroalcoholic solution (growth of the target strains); (ii) broths supplement with either chloramphenicol (for the experiments on bacteria, 200 mg/L) (Sigma-Aldrich, Milan) or cycloheximide (experiments with yeasts, 200 mg/L) (Sigma-Aldrich) (samples with no growth). Both controls were inoculated to 3 or 5 log cfu/mL.

Samples were stored at 25 °C (yeasts and *Pseudomonas* spp.), 30 °C (lactic acid bacteria), and 37 °C (enterobacteria). Microbial growth was evaluated after 24 and 48 h as absorbance at 600 nm with a spectrophotometer UV-VIS DU 640 Beckman (Fullerton, California, USA).

The analyses were performed in duplicate and data were modelled as Growth Index (*GI*) [20]:

(1) $$GI=\left(\frac{Abs_{s}}{Abs_{c}}\right)*100$$

where *Abs_s_* is the absorbance of the samples containing the different amounts of propolis and *Abs_c_* the absorbance of inoculated media containing only hydroalcoholic solution. The analyses were performed in duplicate over two independent batches.

*GI* was read as follows [18]: *GI* < 25%, significant inhibition; 25% < *GI* < 75%, partial inhibition; *GI* > 75%, growth similar to positive control.

For each time of sampling (after 24 or 48 h), *GI* was modelled as a function of propolis concentration through the equation of Weibull, modified by Mafart et al. [21] and cast in the following form:

(2) GI=GI0−(cδ)p

where: *GI* is the Growth index as a function of propolis concentration (dependent variable, %); *GI*_0_, the *GI* of the positive control (broth+hydroalcoholic solution); *c*, the amount of propolis (independent variable, ppm); *δ*, the amount of propolis (ppm) to achieve a reduction of *GI* by 1%; *p*, the shape parameter (dimensionless), which gives some details on the shape of the kinetic: *p* < 1, upward curve; *p* = 1, linear kinetic; *p* > 1, downward curve.

The parameter *δ* was the input for the evaluation of Δ25, that is the amount of propolis required to reduce *GI* by 25% (*δ* × 25). Statistic was performed through the software Statistica for Windows, ver. 7.0 (Statsoft, Tulsa Oklha).

### 2.4. Viable Count of Bacteria and Yeasts

Aliquots of 10 mL of optimal media (YPG broth for yeasts, Nutrient broth for *Pseudomonas* spp and Enterobacteriaceae) were individually inoculated to 5 log cfu/mL with the test strains and added with 100 µL of propolis stock solutions to attain a final concentration ranging between 1000 and 3000 ppm. Two kinds of controls were prepared: (i) media + hydroalcoholic solution (100 µL in 10 mL); (ii) broths supplement with wither chloramphenicol (for the experiments on bacteria, 200 mg/L) or cycloheximide (experiments with yeasts, 200 mg/L). Both controls were inoculated to 5 log cfu/mL.

The samples were stored under static conditions and the growth of the targets was assessed after 24 and 48 h by plate count. The media were the following ones: (i) MRS agar, incubated at 30 °C for 48–72 h under anaerobic conditions for lactic acid bacteria; (ii) YPG agar, incubated at 25 °C for 48 h for yeasts; (iii) Nutrient agar incubated at 25 °C for 48 h for *Pseudomonas* spp. or at 37 °C for 18–24 h for Enterobacteriaceae. The analyses were performed in duplicate over two different batches.

Data were modelled as increase in viable count compared to the inoculum; this value was modelled through MANOVA (multifactorial analysis of variance). The concentration of propolis, the kind of microorganisms and time were used as categorical predictors. The critical *p* was set to 0.05.

### 2.5. Antifungal Activity

The antifungal activity of propolis was tested towards *F. oxysporum* (DSM 2018). The mould was grown on Potato Dextrose Agar (PDA) incubated at 25 °C for 5 days. A conidia suspension was prepared by washing the mould grown on PDA plates with a Tween 80 solution (0.05% *v*/*v*) (C. Erba); conidia suspension (10^7^ conidia/mL) was filtered to avoid the presence of mycelium [22].

After the sterilization, aliquots of stock solutions were added to PDA (55 °C) in order to achieve the desired amount of propolis (1000–1500–2000–2500–3000 ppm) (1 mL of stock solution in 100 mL of medium); PDA + hydroalcoholic solution was used as positive control. Twenty µL of conidia suspension were inoculated in the middle of plates. Incubation was carried out in the dark at 25 ± 2 °C and colony diameters were measured in centimeters and compared with those on controls plates at intervals of 2 days for 10–14 days in order to determine the radial growth.

The experiments were performed on three independent batches; for each batch, the analyses were made in duplicate. Fungal growth was modeled by using a logistic equation, modified by Dantigny et al. [23] and cast in the following form:

(3) $$D=\frac{D\max}{1+\exp[k(\tau -t)]}$$

where *D* is the diameter of the fungal colony over time; *D*_max_, the maximum diameter of fungal colony; *k*, the rate of fungal growth (cm/day); *τ*, the time to attain a ½ of *D*_max_ (day) and *t* the time (day).

## 3. Results and Discussion

Propolis, a natural product of honey bee, has been attracting the attention of researchers due to its various biological activities and therapeutic properties. Flavonoids, aromatic acids, diterpenic acids, and phenolic compounds appear as the principal components that are responsible for the biological activities of propolis samples [24]. Many studies have documented the remarkable action of propolis against viruses, parasites and many types of microorganisms (yeasts, and bacteria) [25].

The first experiment of this research was the evaluation of the antimicrobial activity of different concentrations of propolis through a modified micro-dilution approach; the microorganisms were inoculated at low and high levels and the Growth Index (*GI*) was evaluated, as reported by Bevilacqua et al. [20]. The use of low and high *inocula* was chosen for modelling purposes; low *inocula* (*GI* approach) are useful to build growth kinetics or dose/response curves when the main purpose is to study the amount of antimicrobial at low doses and the expected outcome is a delay of growth rather than a complete inhibition. On the other hand, at higher doses it is suggested to used high *inocula*, because a possible outcome could be a death kinetic.

The microorganisms were chosen for different reasons: enterobacteria are generally the target microorganisms for some foods (for example dairy products), according to EU regulations, while *Pseudomonas* spp. are emerging spoilers, able to start the spoilage of a wide variety of foods [26]. *Lb. plantarum* was used as representative of lactic acid bacteria, because it is widespread in a variety of foods and can be both a technological microorganism (for example starter culture for mozzarella cheese) or a spoiler. Finally, yeasts and *F. oxysporum* were chosen because they can be found in meat (*D. hansenii*), as well as in vegetables or in beverages (*S. cerevisiae* and *F. oxysporum*) [27,28].

*GI* is a tool to point out a significant inhibition due to an antimicrobial compound, if compared to the positive control; it is time-dependent, as it generally changes throughout time if the effect of the antimicrobial compound is reversible.

**Figure 1:**
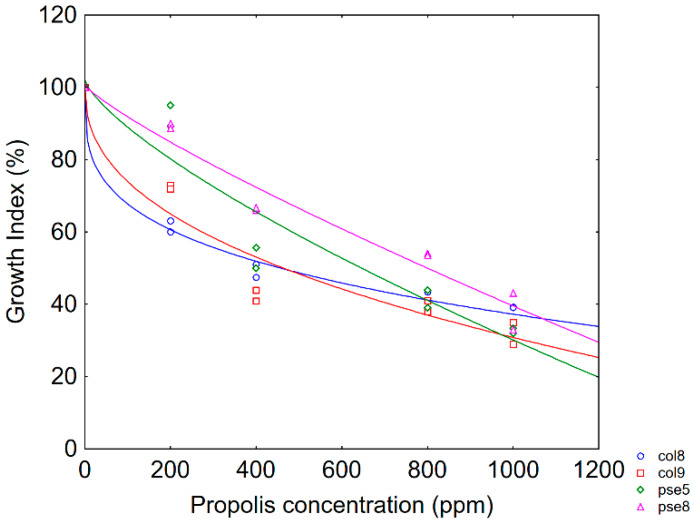
Dose response profile (Growth Index vs. propolis concentration) for pseudomonads and enterobacteria after 48 h. The lines represent the best fitting of data through Weibull model. col8, *H. alvei*; col9, *Enterobacter* spp.; pse5, *Ps. fluorescens*; pse8, *Ps. putida*. Micro-dilution approach for low-level inoculum (3 log cfu/mL).

Therefore, in this paper a static approach was used and for each time of sampling *GI* was modelled as a function of propolis concentration; the use of a static approach (description of a situation for a defined time) was proposed elsewhere as a tool to exclude time-dependence [29]. Generally, *GI* at 48 h were used as input data for modelling except for *S. cerevisiae* (*GI* at 24 h). Figure 1 shows *GI* profile at 48 h for pseudomonads and enterobacteria for low inoculum; all strains experienced an upward linear kinetic with a decrease in *GI* when the propolis amount increased (*GI* was 32%–48% at 1000 ppm), thus suggesting that propolis did not cause a complete inhibition but a significant delay of growth.

**Figure 2:**
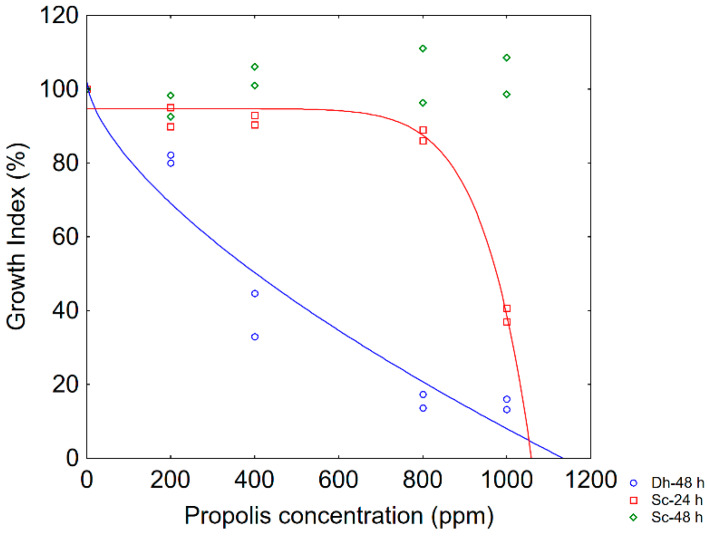
Dose response profile (Growth Index vs. propolis concentration) for yeasts after 24 or 48 h. The lines represent the best fitting of data through the Weibull model. S.c., *S. cerevisiae*; D.h., *D. hansenii*. Micro-dilution approach for low-level inoculum (3 log cfu/mL).

Figure 2 shows the GI profiles for yeasts (low inoculum). *S. cerevisiae* was more resistant than *D. hansenii* as it experienced a downward curve after 24 h; this curve was characterized by a shoulder length of 645 ppm, that is the growth was not affected up to this critical break point. After 48 h, *GI* of *S. cerevisiae* was not affected by propolis, while *D. hansenii* showed an upward curve with a significant negative correlation *GI*/propolis concentration.

The fitting parameters of the Weibull equation and Δ25 are shown in Table 2. Δ25 was strongly species-dependent and was <10 ppm for both enterobacteria; pseudomonads were more resistant, with Δ25 ranging from 77 ppm (*Ps. fluorescens*) to 186 ppm (*Ps. putida*). Finally, Δ25 was 23.50 ppm for *D. hansenii*. Modelling was not performed for *Lactobacillus* spp., because the strains always experienced a *GI* of 90%–100% (data not shown).

When the microorganisms were inoculated at high levels, they never experienced inhibition and the *GI* at 1000 ppm was 76%–89% (data not shown). The *GI* in the samples with antibiotic was always 0%, both at low and high *inocula* (data not shown).

The effect of propolis for the high inoculum level was studied by increasing the amount of the extract; however, a different approach was used (plate count), because a high amount of propolis caused a strong browning of some media (mainly Nutrient broth). The data of viable count were standardized as increase in viable count after 24 or 48 h (ΔC) and analyzed through a multifactorial ANOVA (MANOVA). *Lactobacillus* spp. always experienced a viable count of 9 log cfu/mL and did not show any kind of inhibition; therefore, their data were not used for statistic (data not shown).

**Figure 3:**
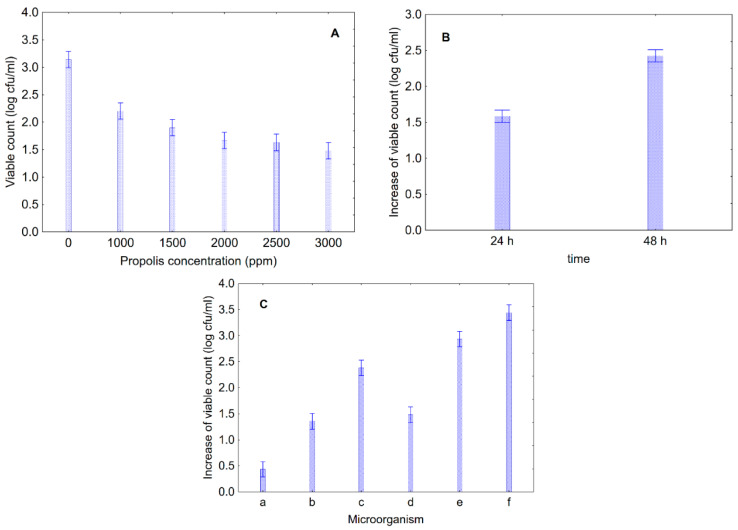
Decomposition of the statistical hypothesis on the increase of viable count of yeast and bacteria. Bars denote 95% confidence intervals. (**A**), effect of propolis concentration; (**B**), effect of time; (**C**), effect of kind of microorganism. a, *D. hansenii*; b, *S. cerevisiae*; c, *H. alvei* (col8); d, *Enterobacter* sp. (col9); e, *Ps. fluorescens*; f, *Ps. putida*.

ΔC was generally positive, thus suggesting that populations increased throughout time. However, MANOVA was affected by both propolis concentration and time, as well as by their interactive terms (propolis concentration*time, time*microorganism, propolis concentration*time*microorganism) (*p* < 0.05). The results of MANOVA are shown in the figures for the decomposition of the statistical hypothesis (Figure 3A–C).

As expected, ΔC was a function of propolis amount (Figure 3A) with a negative correlation; in fact, ΔC decreased when propolis concentration increased, thus suggesting that higher amounts of propolis could be more effective in controlling the growth of some strains. Figure 3B shows the effect of time, with a “false” negative correlation time/bioactivity of propolis, as suggested by the increase in the viable count over time; this effect was due to the fact that propolis generally caused a delay of growth and probably determined an increase in the lag phase or a reduction in growth rate in the bacterial or yeast-kinetic.

Finally, Figure 3C shows the effect of the kind of microorganism; this figure could be used to point out a kind of resistance hit: *D. hansenii* was less resistant than *S. cerevisiae*. Among bacteria, *Pseudomonas* spp. were more resistant than enterobacteria; for enterobacteria, *H. alvei* was more resistant than *Enterobacter* sp.

Concerning the anti-yeast effect, De Castro et al. [30] suggested that 0.125% (1250 ppm) propolis could be an adequate choice as a sub-inhibitory concentration for *S. cerevisiae*, thus confirming the strong resistance of this species as found both for *GI* and viable count. The authors suggested a dual role for propolis treatment as an agent that induces apoptosis and secondary necrosis; in addition, propolis inhibited respiration in *S. cerevisiae*. Regarding *D. hansenii*, Sidra [31] reported 0.4 and 0.8 mg/mL (ranging from 400 to 800 ppm) as the Minimum Inhibitory Concentrations (MIC) in orange and apple juice, respectively.

For the antibacterial effect of propolis, it is generally assumed that that Gram positive bacteria are sensitive to low propolis concentration and Gram-negative bacteria could be only inhibited with higher propolis dose [25]; therefore, few data are available on Gram negative and some results could be found on pathogens (*Yersinia enterocolitica*, *Ps. aeruginosa*, *Escherichia coli*, *Salmonella* sp., etc.) [32,33,34,35]. However, to the best of our knowledge, few data are available on the spoilers, such as *Pseudomonas* spp., which are a challenge for food producers.

It has been suggested that the resistance of Gram-negative bacteria could be due to the presence of efflux pumps preventing the intracellular entry of propolis constituents. The weak effect on Gram-negative bacteria may also be explained by the fact that propolis contains mainly plant-derived resin constituents and that resins are secreted by plants to mostly protect from Gram-positive pathogens [36].

The data of this research suggest that propolis could be used for the inhibition of Gram-negative bacteria, although pseudomonads require medium-to high levels, thus confirming the resistance of pseudomonads to extracts [37,38]. Some authors studied the mode of action of propolis towards *Ps. aeruginosa* and found that propolis impaired the growth, the production of biofilm and the capacity to release molecules, such as phenazines and eDNA with a possible role of complex phenols [39].

Although the effect of propolis on Gram negative was reported as low or moderate, the data of this research showed a strong effect on the delay of growth kinetic for enterobacteria, due probably to a higher sensitivity of the tested strains. For these microorganisms, multiple effects could be responsible for the antibacterial activity, including the inhibition of cell division, and protein synthesis [9].

Another effect found on the tested target was the dependence of the antimicrobial effect on the cell concentration, as at high inoculum, an effect of propolis was found only at high doses. This is a new evidence and further investigations are required. Finally, the resistance of *Lb. plantarum* to propolis was probably the result of the high resistance of this species to phenols (complex or simple phenolic compounds) reported elsewhere by the authors [40].

**Figure 4:**
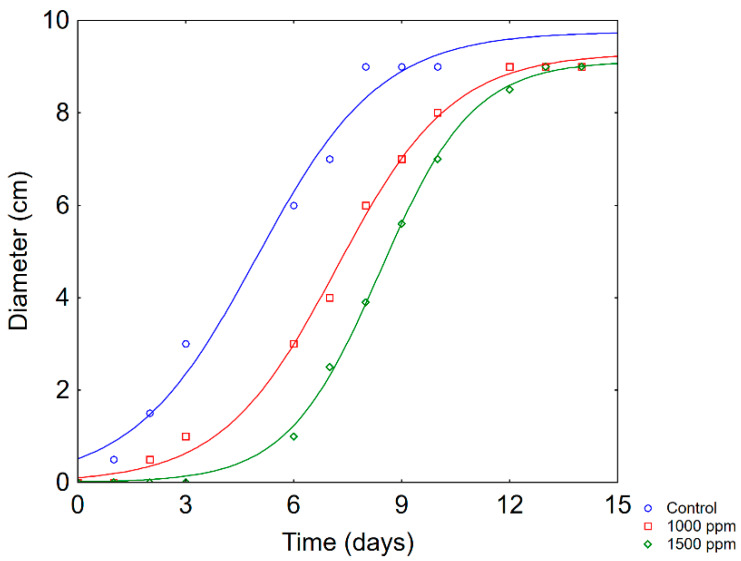
Growth kinetic of *F. oxysporum* on plates as a function of propolis concentration; the points represent the average of three determinations. Lines are the best fit through Dantigny model.

The antifungal effect of propolis was tested towards *F. oxysporum* by assessing the radial growth on plates. Figure 4 shows some examples of fungal kinetics; they generally experienced a logistic-like shape, characterized by a three-phase kinetic (a lag phase, an exponential growth phase and a steady state); thus, they were modelled through a logistic function (the equation of Dantigny et al. [23]).

For fungi, the fitting parameters of a sigmoid have a different meaning if compared to a bacterial kinetic; Dantigny function has a main parameter, that is the time to attain a ½ of the maximum diameter (*τ*), which is an estimation of both the first step (no growth) and fungal growth rate (exponential phase) (Table 3). *τ* was 4.98 ± 0.44 days in the positive control and increased to 7.20 ± 0.11 days on PDA + 1000 ppm propolis and 8.40 ± 0.06 on PDA + 1500 ppm propolis; a further increase of propolis did not significantly act on *τ*.

The results on the antifungal activity are generally in line with the literature. According to AL-Ani et al. [25], propolis possess a moderate antifungal activity. Ôzcan [41] reported that the concentration of 4% of propolis extract is able to inhibit *F. oxysporum* f. sp. *melonis* by 50%. In another study [42], 5 mg/mL (5000 ppm) of ethanol extract of propolis completely inhibited the radial growth of *F. oxysporum* on solid media (PDA). Various compounds like phenolics and flavonoids are responsible for their antifungal activity by affecting the permeability of the cytoplasmic membrane, which leads to the total leakage of the cellular constituents such as nucleic acids, proteins and inorganic ions such as phosphate and potassium, leading to complete cell death [43].

Generally, the effects of propolis resulted in a delay of fungal or bacterial growth, as suggested by the reduction in *GI* as well as by the increase in τ for *F. oxysporum*; however, further investigations are required to elucidate some key-points related to food applications. An open question is the evaluation of MIC, NIC (Not Inhibitory Concentration) [44] and the dose/response profile. The results of this paper suggest a MIC > 3000 ppm, with an NIC ranging from 10 ppm (reduction in GI by 25% for enterobacteria) to 645 ppm (shoulder length of *S. cerevisiae*). The high MIC suggests a possible application in designing food-packaging materials or edible coatings, rather than use as ingredients in food formulas. In fact, the high doses required for propolis bioactivity are not compatible for a direct application, due to the possible strong impact on the organoleptic characteristic of foods.

On the other hand, the filming properties of propolis [45,46], along with the delay of some spoilers as suggested in this paper, could be the background to design an active packaging for dairy products, meat, or vegetables.

## 4. Conclusions

The antimicrobial effect of propolis relies upon several factors, and this research contributed to point out some of them. First, the effect was strongly related to the kind of microorganisms; for yeasts, *D. hansenii* was more affected than *S. cerevisiae*. Concerning bacteria, *Lb. plantarum* was never affected, while propolis controlled the growth of both *Pseudomonas* and Enterobacteriaceae. Another significant effect was related to cell concentration, because at high inoculum no (or very mild) effect was found; finally, propolis delayed the radial growth of *F. oxysporum*, as suggested by the increase in the parameter *τ*. The results of this paper point out, for the first time, the antimicrobial activity of propolis against some food spoilers, with a focus on the possible effect (that is, the delay of growth rather than a complete inhibition). However, further investigations are required to point out an exact definition of the dose response/curve of propolis (MIC), as a prodromal step for a possible application in food industry (packaging, new edible coatings).

## Figures and Tables

**Table 1:** Microorganisms.

Target	Source
*Pseudomonas putida* (PSE8)	Wild strain isolated from mozzarella cheese
*Pseudomonas fluorescens* (PSE5)	Wild strain isolated from mozzarella cheese
*Hafnia alvei* (COL8)	Wild strain isolated from mozzarella cheese
*Enterobacter* spp. (COL9)	Wild strain isolated from mozzarella cheese
*Lactobacillus plantarum* (L12)	Wild strain isolated from sourdough
*Lactobacillus plantarum* DSM1055 *	Collection strain
*Debaryomyces hansenii* DSM3428 *	Collection strain
*Saccharomyces cerevisiae* EC1118 **	Commercial wine strain
*Fusarium oxysporum* DSM2018 *	Collection strain

* Deutsche Sammlung von Mikroorganismem und Zellkulturen’s collection- Braunschweig, Germany; ** Lallemande (Canada).

**Table 2:** Parameters of Weibull equation: *δ*, amount of propolis (ppm) to achieve a reduction in Growth Index of 1%; *p*, shape parameter (*p* < 1, upward curve; *p* > 1, downward curve); Δ25, amount of propolis (ppm) to achieve a reduction of Growth Index of 25%; R^2^, determination coefficient. Micro-dilution approach for low-level inoculum (3 log cfu/mL).

Microorganism	Time	*δ*	*p*	Δ25	R^2^
*D. hansenii*	48 h	0.94 ± 0.11	0.65 ± 0.09	23.50	0.935
*S. cerevisiae*	24 h	645.73 ± 69.04	9.20 ± 2.10	/†	0.977
	48 h	-	-	-	-
*Ps. fluorescens*	48 h	3.07 ± 0.22	0.74 ± 0.05	76.75	0.909
*Ps. putida*	48 h	7.44 ± 1.25	0.84 ± 0.01	186.00	0.962
*H. alvei*	48 h	0.01 ± 0.01	0.29 ± 0.01	<10	0.990
*Enterobacter* sp.	48 h	0.04 ± 0.01	0.42 ± 0.03	<10	0.941

† Not evaluated.

**Table 3:** *F. oxysporum τ* (time to attain a ½ of the maximum diameter) (day) on PDA + propolis (5–15%). Mean values ± standard error. R^2^, determination coefficient of Dantigny model. The letters indicate significant differences (one-way ANOVA and Tukey’s test, *p* < 0.05).

Propolis Amount	*τ*	R^2^
0 (control)	4.98 ± 0.44a	0.985
1000 ppm	7.20 ± 0.11b	0.997
1500 ppm	8.40 ± 0.06c	0.999
2000 ppm	8.33 ± 0.08c	0.998
2500 ppm	8.42 ± 0.08c	0.998
3000 ppm	8.29 ± 0.09c	0.997
